# Management of brain metastases in lung cancer: evolving roles for radiation and systemic treatment in the era of targeted and immune therapies

**DOI:** 10.1093/noajnl/vdab106

**Published:** 2021-11-27

**Authors:** Nathaniel J Myall, Helena Yu, Scott G Soltys, Heather A Wakelee, Erqi Pollom

**Affiliations:** 1 Division of Oncology, Department of Medicine, Stanford Cancer Institute, Palo Alto, California, USA; 2 Department of Medicine-Oncology, Memorial Sloan Kettering Cancer Center, New York City, New York, USA; 3 Department of Radiation Oncology, Stanford Cancer Institute, Palo Alto, California, USA

**Keywords:** brain metastases, immunotherapy, lung cancer, radiotherapy, targeted therapy

## Abstract

Brain metastases are a common occurrence in both non-small cell and small cell lung cancer with the potential to affect quality of life and prognosis. Due to concerns about the accessibility of the central nervous system by systemic chemotherapy agents, the management of brain metastases has historically relied on local therapies including surgery and radiation. However, novel targeted and immune therapies that improve overall outcomes in lung cancer have demonstrated effective intracranial activity. As a result, the management of brain metastases in lung cancer has evolved, with both local and systemic therapies now playing an important role. Factors such as tumor histology (non-small versus small cell), oncogenic driver mutations, and symptom burden from intracranial disease impact treatment decisions. Here, we review the current management of brain metastases in lung cancer, highlighting the roles of stereotactic radiosurgery and novel systemic therapies as well as the ongoing questions that remain under investigation.

Brain metastases are the most common adult intracranial tumor, and their incidence is increasing, driven largely by lung cancer.^1^ As the most common cancer to develop brain metastases, lung cancer presents with intracranial involvement in approximately 20% of patients at the time of diagnosis.^2^ This increased incidence may be due to both improved neuroimaging with more frequent use of MRI and improved prognosis from more effective systemic therapy. Thus, surveillance brain imaging is routinely recommended for patients with newly diagnosed lung cancer, regardless of symptoms.

The morbidity of brain metastases is related to both the direct neurological complications of the disease as well as the side-effects of local therapy. Depending on the size, location, and extent of edema associated with brain metastases, symptoms can include headache, altered mental status, focal motor or sensory deficits, and ataxia. Although the prognosis of patients with lung cancer brain metastases has historically been poor, outcomes are improving with median survival now reaching 12 months overall and 15 months in patients with adenocarcinoma in particular.^3^ The Graded Prognostic Assessment (GPA) index can help predict prognosis in patients with brain metastases based on age, performance status, number of brain metastases, and the presence of extracranial disease.^4^ This GPA index has also been updated to incorporate gene alteration data including *EGFR* and *ALK* mutations in a modified prognostic index (Lung-molGPA), with the highest scores corresponding to a median survival of nearly 4 years.^5^

The treatment of brain metastases in lung cancer combines both local (eg, radiation, surgery) and systemic therapies. In this review, we summarize the available literature supporting these therapeutic interventions and describe our evidence-based approach to the management of brain metastases in both non-small cell and small cell lung cancer.

## Non-Small Cell Lung Cancer

**Figure 1.**
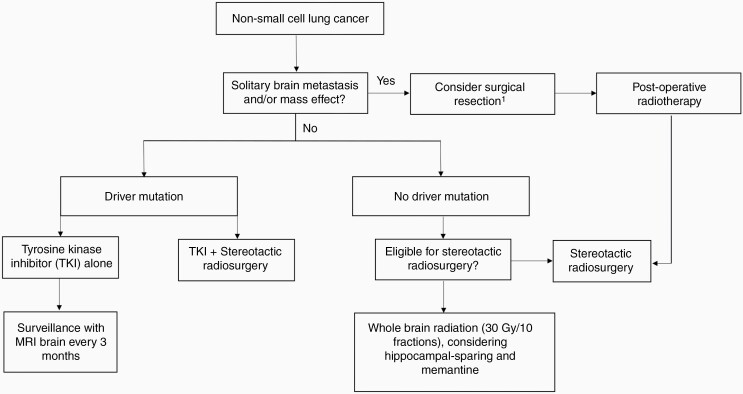
Approach to the management of brain metastases in patients with non-small cell lung cancer. ^1^Decisions regarding surgery should be considered only in carefully selected patients in a multidisciplinary setting.

The systemic treatment of non-small cell lung cancer (NSCLC) has advanced significantly over the last decade due to the emergence of tyrosine kinase inhibitors (TKI) and immunotherapy. Although the central nervous system (CNS) has been classically considered a protected site due to the presence of the blood-brain barrier, these novel therapies have good intracranial penetration and are changing the management of intracranial disease in lung cancer. In addition, radiotherapy strategies have evolved, with stereotactic radiosurgery (SRS) supplanting whole-brain radiotherapy (WBRT) in most circumstances. As a result, the management of patients with intracranial metastases from NSCLC remains complex and requires multidisciplinary care that takes into account the presence of oncogenic driver alterations, the size, number, and volume of intracranial lesions, and the extent of extracranial disease (Figure 1).

### Indications for Surgical Resection

For patients with large parenchymal brain metastases with symptomatic mass effect and reasonable performance status and prognosis, or when there is diagnostic uncertainty, surgical resection is the preferred initial treatment. Surgery may also be considered as one part of a definitive, multi-modality treatment approach for patients who present with oligometastatic NSCLC in the form of solitary brain metastasis. In an older, randomized trial of patients with single brain metastasis, the majority of whom had lung cancer and no evidence of disease outside of the brain and primary site, the addition of surgical resection to WBRT significantly prolonged overall survival.^6^ A more recent prospective study of patients with oligometastatic NSCLC, 44% of whom had brain involvement, reported that the addition of definitive surgery or radiation to both primary and oligometastatic disease sites resulted in a 3-year overall survival rate of 17.5%, which compared favorably to historical controls.^7^ Factors associated with improved outcomes in this setting include lower primary (T) and lymph node (N) stage within the lung and concurrent definitive management of the primary tumor with complete resection or radiation.^8–11^ Therefore, any decision to pursue an aggressive, definitive-intent surgical approach for an asymptomatic, solitary brain metastasis must carefully consider a patient’s overall stage of disease and his or her fitness for treatment including surgery and chemotherapy.

Following surgical resection of brain metastases, postoperative radiation is recommended to reduce the risk of local recurrence. WBRT had previously been the standard treatment as it addresses both gross and microscopic intracranial disease and decreases the risk of local recurrence, distant intracranial recurrence, and neurologic death.^12,13^ However, the role of WBRT has declined due to advances in systemic therapy as well as radiotherapy delivery. Image-guided systems have enabled stereotactic radiosurgery (SRS), which allows the delivery of high doses of radiation to small targets with high precision. In addition to the improved side effect profile compared to WBRT, the ablative doses of SRS improve local tumor control, particularly for radioresistant histologies. Reducing long-term neurotoxicity and local progression is increasingly important in the setting of improved systemic therapy and survival in patients with lung cancer. Post-operative SRS to the resection cavity has a less deleterious cognitive impact than WBRT in the adjuvant setting and is, therefore, the standard of care for patients with limited brain metastases following resection.^14^ Further details of radiation modalities and techniques are well-covered in other articles in this issue (“Advances in radiation therapy for brain metastases”).

### Approach to NSCLC without a Driver Mutation

Patients with brain metastases from NSCLC without a driver mutation are managed similarly to patients with brain metastases from other primary sites.

***SRS and WBRT*.**—Patients with small, asymptomatic brain metastases can be treated with radiation alone without resection. As in the adjuvant radiation setting, SRS is increasingly used for intact brain metastases to balance treatment toxicities with intracranial control. For patients with a limited number of brain metastases, SRS is preferred as it has improved cognitive outcomes compared to WBRT, with no difference in survival.^15^ While SRS trials have included patients with up to 3–4 brain metastases, there are accumulating prospective data supporting SRS for patients with up to 10–20 brain metastases.^16,17^ Additionally, total intracranial tumor volumes are better correlated with survival than is the number of brain metastases.^18^ However, SRS is not without risk and can result in the development of post-treatment radiation necrosis given the high doses of radiation used. Radiation necrosis is an inflammatory reaction around an irradiated lesion that can be symptomatic and may require management with steroids, bevacizumab, and/or resection.

For patients with brain metastases who are not candidates for radiosurgery, WBRT with hippocampal sparing and memantine to reduce the adverse impact of radiation on quality of life and neurocognition is the new standard of care.^19^ Patients with guarded prognosis and poor performance status, however, may derive little benefit from WBRT. The Quality of Life after Treatment for Brain Metastases (QUARTZ) trial randomized patients with advanced NSCLC to best supportive care including dexamethasone with or without WBRT. There was no clinically meaningful benefit in terms of quality of life or survival with the addition of WBRT. Notably, patients on this trial had a median survival of only 2 months.^20^ Subset analyses suggested improved survival with WBRT for younger patients with higher performance status and controlled primary disease. Thus, for certain patients with limited expected survival, the best supportive care is an acceptable alternative.

For patients with leptomeningeal disease, WBRT is an important palliative treatment option. Although WBRT has not been shown to improve survival in this clinical scenario, its effect on symptoms or quality of life was not evaluated.^21^ Leptomeningeal disease can cause significant symptoms including cranial and spinal neuropathies and headaches due to elevated intracranial pressure and hydrocephalus. Focal radiation can be directed to symptomatic, bulky disease in the brain or spine and can lead to symptom improvement. While irradiation of the entire craniospinal axis is not typically recommended due to concern for significant myelosuppression and neurotoxicity, newer techniques using protons can potentially improve the tolerability of craniospinal irradiation.^22^ Intrathecal methotrexate and cytarabine have also been studied in leptomeningeal disease from solid tumors but are only modestly effective and are limited by side-effects including chemical meningitis.^23^ Additionally, systemic therapies with intracranial activity, such as pemetrexed, bevacizumab, and targeted therapies, now offer better-tolerated, potentially more effective alternatives beyond intrathecal chemotherapy and WBRT, as discussed below.^24^

Finally, prophylactic cranial irradiation (PCI) has also been explored for NSCLC but unlike in small cell lung cancer (SCLC), most studies have failed to show a survival benefit with the addition of PCI.^25–28^ RTOG 0214 randomized 356 patients with stage III NSCLC to PCI or observation following initial therapy and found that PCI significantly reduced the incidence of brain metastases (1 year 7.7% versus 18.0 %) but did not improve overall or disease-free survival.^28^ The long-term update of this trial did find a survival benefit among patients who did not undergo surgery, suggesting that perhaps certain patient subgroups could benefit long-term from PCI.^27^ A recent phase 2 randomized trial of PCI among 84 patients with Stage IIIB or IV NSCLC at high risk for developing brain metastases (adenocarcinoma subtype with targetable mutation and elevated CEA) did show a survival benefit (64.5 versus 19.8 months, *P* = .007).^29^ It is important to note, though, that patients on this study did not have access to third-generation TKIs which have been proven to be highly effective at decreasing the incidence of brain metastases as discussed later in this review. More study is warranted of the benefit of PCI in NSCLC in the setting of newer systemic therapies and in specific patient populations before this treatment can be adopted into standard practice.

***Non-targeted systemic therapy: chemotherapy, immunotherapy & bevacizumab*.**—Chemotherapeutic agents have traditionally had limited intracranial activity. However, most patients with brain metastases will have concurrent extrathoracic disease that requires systemic therapy. In patients whose tumors lack an oncogenic driver mutation, chemotherapy typically forms the backbone of such therapy. Although several studies did not show a survival benefit with the addition of chemotherapy to radiation in patients with NSCLC brain metastases and limited extracranial disease, these trials primarily evaluated chemotherapy regimens that are not widely used today for the systemic treatment of NSCLC.^30,31^ On the other hand, studies of platinum-doublet chemotherapy have demonstrated comparable overall survival and, in some cases, intracranial response rates in patients receiving chemotherapy alone with radiation reserved for the time of progression versus concurrent upfront chemotherapy plus radiation.^32–34^

Pemetrexed is an antimetabolite used in combination with cisplatin or carboplatin for non-squamous cell lung cancer that has shown encouraging intracranial activity. In a retrospective analysis of two-phase 3 trials of metastatic NSCLC, the incidence of brain-only metastases at the time of progression was significantly lower in patients receiving pemetrexed compared to non-pemetrexed chemotherapy (3.2% vs. 6.6%).^35^ Consistent with pemetrexed’s histology-specific activity, the rate of brain-only metastases at progression was significantly lower in patients with non-squamous cell lung cancer whereas no difference was seen between pemetrexed and other regimens in patients with squamous cell lung cancer. In a prospective study of patients with NSCLC presenting with asymptomatic brain metastases, the combination of cisplatin plus pemetrexed was associated with an intracranial objective response rate (ORR) of 42%.^36^ However, all patients also received WBRT following up to 6 cycles of cisplatin plus pemetrexed.

Immune checkpoint inhibitors targeting the PD-1/PD-L1 and CTLA-4 axes are now routinely used alone or in combination with chemotherapy as first-line treatment of metastatic NSCLC that does not harbor an *EGFR* mutation or *ALK/ROS1* rearrangements. In a recent phase 2 trial assessing the intracranial activity of pembrolizumab monotherapy (PD-1 inhibitor) in patients with NSCLC presenting with asymptomatic, untreated, or progressing intracranial metastases measuring 5–20 mm in size, the intracranial ORR was 30% in patients with PD-L1 expression ≥1%.^37^ In comparison, no responses were seen in a second, smaller cohort of patients whose tumors were negative or not evaluable for PD-L1. In a separate phase 2 trial of pembrolizumab monotherapy in patients with leptomeningeal metastases from multiple cancer histologies, stable intracranial disease was reported in 55% of the heavily pretreated cohort according to Immunotherapy Response Assessment in Neuro-Oncology (iRANO) criteria.^38^ While this suggests possible activity for more advanced intracranial disease, only one patient in this cohort had NSCLC, and further study of leptomeningeal disease is therefore necessary.

Despite these findings, the brain is still a frequent site of recurrence in patients receiving checkpoint inhibitor therapy. Follow-up analysis of the PACIFIC trial, for example, showed that among patients with extrathoracic disease recurrence, the brain was a site of involvement in 62% of patients receiving durvalumab versus 67% of those receiving placebo.^39^ In addition, studies of pembrolizumab in patients with melanoma suggest that salvage local therapy is frequently required for lesion growth, edema, or hemorrhage even in patients not receiving upfront radiation for lesions measuring 5–20 mm in size.^40^ Because studies to-date evaluating pembrolizumab in patients with intracranial metastases have had strict inclusion criteria, their findings are also of limited generalizability to patients with larger and/or symptomatic lesions. Consistent with the hypothesis that radiation may augment immune functions such as antigen presentation and T-cell activation, retrospective studies have also suggested a local control and survival benefit for concurrent checkpoint inhibition plus SRS in lung cancer and other malignancies.^41,42^ While these studies showed combined checkpoint inhibition and intracranial radiation to be safe without an increased incidence of radiation necrosis or other neurological complications, others have suggested increased rates of radiation necrosis with the combination approach.^43^ Patients receiving immunotherapy may also develop a transient enlargement of brain metastases following SRS, termed “pseudoprogression”, which may be symptomatic or mistaken for true progression.^44^ Therefore, as with any other novel therapy, prospective studies are eagerly awaited to define the role and safety of concurrent checkpoint inhibition and radiotherapy and identify predictive factors for their combined use in patients with intracranial metastases.

Bevacizumab, a monoclonal antibody targeting vascular endothelial growth factor (VEGF), has also been studied in patients with brain metastases in combination with both radiotherapy and other systemic agents. Although early trials excluded patients with brain metastases due to concerns about the risk of intracerebral hemorrhage, subsequent analyses showed bevacizumab to be safe in patients with brain metastases from solid tumors.^45–47^ This paved the way for the phase 2 BRAIN trial, which demonstrated similar rates of response to carboplatin, paclitaxel, and bevacizumab in both intracranial and extracranial lesions (61% vs. 64%, respectively) in patients with untreated, asymptomatic brain metastases from NSCLC.^48^ In a retrospective analysis of the AVAiL trial, the addition of bevacizumab to cisplatin plus gemcitabine in patients without baseline intracranial disease was also associated with a significant reduction in the incidence of intracranial metastases as site of first progression (2.6% vs. 5.8%).^49^ Similarly, despite not meeting the trial’s primary endpoint, the addition of bevacizumab to adjuvant chemotherapy in the ECOG-ACRIN E1505 trial was associated with a lower risk of brain metastases as the first site of recurrence in subset analyses.^50^

On the other hand, the addition of bevacizumab to erlotinib in the NEJ026 trial did not improve PFS compared to erlotinib alone in the subset of patients with baseline brain metastases.^51^ In addition, the role of bevacizumab in metastatic NSCLC overall continues to evolve now that targeted therapies and immune checkpoint inhibitors occupy clearly defined roles in the metastatic setting. Therefore, while bevacizumab appears to be safe in combination with intracranial radiation in metastatic solid tumors, further work is needed to determine the most effective and optimal use of bevacizumab in patients with lung cancer intracranial metastases.^52^

Overall, pemetrexed, pembrolizumab, and bevacizumab do not replace the need for local radiotherapy. However, the added intracranial activity with each of these systemic therapies has the potential to improve overall outcomes in combination with radiotherapy. This is particularly important in patients without targetable oncogenic driver mutations who lack oral TKI therapy options and remain at risk for developing intracranial progression during the course of their disease.

### Approach to NSCLC with a Driver Mutation

***EGFR-targeted therapies***.—Patients with a driver mutation will respond to many of the brain-penetrable TKI therapies, which may allow deferral of local therapy for asymptomatic brain metastases. Although this strategy has not been tested in randomized trials, third-generation TKIs such as osimertinib can achieve high intracranial concentrations^53^ and have shown intracranial response rates of up to 91% (Table 1).^54^ Although retrospective studies have suggested potentially worse survival with deferral of radiotherapy for patients with brain metastases from *EGFR*-mutant lung adenocarcinoma receiving TKI therapy, these studies included only first- or second-generation EGFR TKIs which have intracranial activity inferior to osimertinib.^55,56^ In contrast, a more recent retrospective, multi-center study of patients with *EGFR*-mutant NSCLC receiving CNS-penetrant TKI therapy (osimertinib or rociletinib) for new or progressing brain metastases found no statistically significant difference in time to overall progression or time to intracranial progression in patients receiving TKI therapy with or without radiotherapy.^57^ These results suggest that deferral of upfront radiation may be a safe strategy for carefully selected patients receiving newer-generation therapies, particularly if brain metastases are small and asymptomatic. However, to what extent lesion size, number of metastases, and/or presence of symptoms should impact therapeutic decision-making remains unclear. Additionally, deferral of radiotherapy may also not be appropriate for patients with limited oligometastatic disease for whom an aggressive local ablative approach is considered. Prospective confirmation is therefore awaited, with the phase II OUTRUN trial (NCT03497767) currently underway to evaluate osimertinib versus osimertinib plus SRS in patients with ≤10 intracranial metastases. In the meantime, while the optimal combination and sequencing of TKI and SRS and impact of treatment strategies on outcomes such as quality of life remain to be clarified prospectively, patients who are managed with TKI alone and deferral of radiotherapy upfront should get short-interval imaging to confirm the response of intracranial disease.

**Table 1.** Intracranial Activity of Current First-Line EGFR and ALK Targeted Inhibitors

Trial	Therapy	iORR^a^ (Measurable Disease)	iORR (Measurable or Non-measurable)	iPFS at 12- or 24-months^b^	Cumulative Incidence of CNS Progression^c^
FLAURA^54^	Osimertinib	91%	66%	77% (12 mos)	8%
ALEX^58,59^	Alectinib	81%	59%	-	9%
ALTA-1L^60,61^	Brigatinib	78%	67%	48% (24 mos)	-
CROWN^62^	Lorlatinib	82%	66%	-	3%

^a^iORR, intracranial objective response rate.

^b^iPFS, intracranial progression-free survival.

^c^Assessed at 12-months.

***ALK-rearrangements and other oncogenic drivers***.—Although EGFR serves as the paradigm for driver mutated lung cancer, brain metastases are also frequently seen in lung cancers harboring less common targetable alterations such as *ALK*, *ROS1*, or *RET* rearrangements or *BRAF*, *HER2*, or *MET* exon 14 skipping mutations.^63–68^ In *ALK*-rearranged lung cancer, three FDA-approved, first-line inhibitors (alectinib, brigatinib, and lorlatinib) demonstrate high intracranial response rates ranging from 78–82% in patients with measurable intracranial disease (Table 1).^58–62^ While each of these agents has shown superior activity relative to crizotinib, none have been compared to the others directly, and cross-trial comparisons should be made with caution. Therefore, selection between these first-line options should consider other factors such as their side-effect profiles and clinician familiarity rather than head-to-head comparisons of intracranial response rates from across separate trials. In addition, data suggesting lorlatinib activity against particular secondary *ALK* resistance mutations (eg, *ALK* G1202R) may provide a rationale for using lorlatinib in the relapsed, refractory setting regardless of baseline CNS metastases.^69^

As with *EGFR*-mutant lung cancer, a remaining question with highly effective ALK inhibitors is when to use them alone versus in combination with radiotherapy for intracranial metastases at diagnosis. In a secondary analysis of the ALEX trial, the superiority of alectinib over crizotinib was maintained regardless of prior intracranial radiotherapy, with comparable intracranial response rates among patients with measurable CNS disease who did and did not receive prior radiotherapy (86% vs. 79%).^59^ However, the cumulative incidence rate (CIR) of intracranial metastases was lower in those patients with baseline CNS metastases who received alectinib plus radiotherapy compared to alectinib alone (12-month CIR 9% vs. 21%, no *P*-value given), suggesting that long-term intracranial disease control may be improved with radiotherapy compared to the use of ALK-directed TKI therapy alone. In one multi-center retrospective analysis, neither time to overall progression nor time to intracranial progression was statistically different in patients receiving alectinib, lorlatinib, brigatinib, or ensartinib alone versus in combination with radiotherapy.^57^ However, among those patients receiving radiation, there was a trend toward having larger and/or symptomatic intracranial metastases. Therefore, ongoing studies are needed to define which patients with *ALK*-rearranged NSCLC in the era of brain-penetrable TKI therapy will continue to benefit from upfront radiotherapy due to being at higher risk for intracranial progression.

Brain-penetrable therapies are also emerging for other infrequent driver mutations. In *ROS1*-rearranged NSCLC, lorlatinib had an intracranial response rate of 64% in patients who were TKI-naïve.^70^ Capmatinib and tepotinib are both active in lung cancer harboring *MET* exon 14 skipping mutations, with associated intracranial response rates of ~50%.^71,72^ Finally, selpercatinib has been reported to have an intracranial response rate of 91% in patients with *RET*-fusion-positive NSCLC.^73^ In many of these trials, however, the number of patients with evaluable CNS disease was small, and therefore, as these and other novel targeted therapies continue to be studied, intracranial activity should remain an important benchmark in clinical trials.

***Leptomeningial disease***.—Patients with driver-mutated lung cancer are also at risk of leptomeningeal metastases during their disease course. For *EGFR*-mutant NSCLC, the incidence of leptomeningeal disease has been reported to be higher than that of wild-type *EGFR* cohorts (9.4% versus 1.7%).^74^ Fortunately, osimertinib appears to be active in this setting as described in the phase I BLOOM trial, which reported a leptomeningeal ORR of 62% according to RANO criteria and complete cytologic clearance from the CSF in 28% of patients receiving osimertinib 160 mg daily.^75^ Notably, response rates were numerically similar (55% versus 57%) in patients receiving or not receiving prior intracranial radiotherapy. Whether high-dose osimertinib is required to achieve intracranial concentrations sufficient to treat leptomeningeal disease has come into question as standard-dose osimertinib 80 mg daily has also shown activity in a retrospective series and may therefore represent a better-tolerated option.^76^ Collectively, these results suggest that osimertinib represents a potential radiation-sparing treatment option in patients with *EGFR*-mutant lung cancer metastatic to the leptomeninges. Prospective trials of other brain-penetrable TKI therapies targeting driver mutations beyond EGFR are needed to confirm whether a similar approach can be taken in these patients with leptomeningeal disease as well, as has been suggested by early case reports.^77^

***Post-progression management after TKI therapy.***—Patients managed with TKIs can also develop isolated intracranial progression while on therapy. It is unclear whether this is due to acquired resistance versus the brain being a “sanctuary” site. This underscores the need for brain-penetrable TKI therapies that not only effectively treat existing intracranial metastases but also lower the cumulative incidence of subsequent metastases. For patients who develop new or progressing intracranial metastases on TKI therapy, radiotherapy at the time of progression remains an effective option enabling continued use of post-progression TKI therapy.^78^ Alternatively, the systemic therapies described above for patients without a driver mutation (see “Non-Targeted Systemic Therapy: Chemotherapy, Immunotherapy & Bevacizumab”) can also be considered in the later-line setting following TKI therapy especially if there is concurrent extracranial progression as well. Some TKI therapies such as osimertinib have also been safely used in combination with carboplatin-doublet chemotherapy.^79^ The choice to continue TKI therapy while also adding chemotherapy may be particularly advantageous in patients who experience extracranial progression but are considered to have ongoing control of intracranial disease from their TKI therapy.

***Future directions: genetic drivers of brain metastases.***—Driver alterations such as those occurring in *EGFR* and *ALK* are responsible for tumorigenesis and therefore represent early, seminal genetic events in lung cancer development. Branched evolution of lung tumors over time, however, may lead to the development of additional genetic alterations that differ between metastases and primary tumors, thereby contributing to genetic heterogeneity across sites of disease.^80^ In the case of lung adenocarcinoma, sequencing analyses comparing primary tumor versus metastatic brain sites have identified alterations that are more common in brain metastases such as amplification of *MYC* and mutations in *TP53*.^81,82^ If such alterations underlie the ability of tumors to metastasize to the brain, development of therapies targeting these alterations could eventually enable a systemic treatment approach that is specifically tailored to the genetic environment driving lung cancer brain metastases.

## Small Cell Lung Cancer

**Figure 2.**
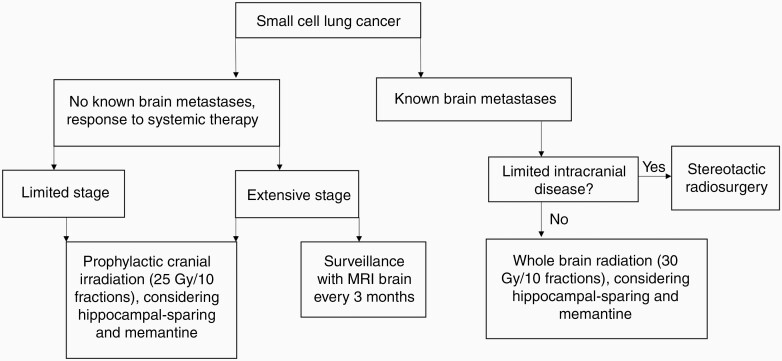
Approach to the management of brain metastases in patients with small cell lung cancer.

Small cell lung cancer differs from NSCLC in that its natural history reflects more rapid growth and dissemination. Given concern for high rates of occult brain metastases among patients with SCLC, brain management has typically included WBRT for patients with known brain metastases and PCI for patients without known brain metastases. However, this paradigm has been shifting with improved imaging surveillance and radiation technology (Figure 2).

### Prophylactic Cranial Irradiation

PCI has been studied in the randomized setting in both limited-stage (LS) and extensive stage (ES) SCLC. A meta-analysis of 7 randomized trials showed that among patients with LS-SCLC who achieved a complete remission to chemotherapy, PCI decreased the incidence of brain metastases by almost half and improved 3-year survival by 5%.^83^ Patients with ES-SCLC treated with PCI following chemotherapy also had decreased incidence of symptomatic brain metastases and increased survival on the EORTC trial.^84^ However, these trials were conducted in an era prior to routine brain imaging. A more recent trial from Japan of patients with ES-SCLC who had a response to chemotherapy required brain MRIs prior to randomization and randomized patients to PCI or active surveillance with brain MRIs every 3 months during the first year followed by every 6 months during the second year.^85^ The trial was stopped early due to futility. While PCI did decrease incidence of brain metastases (1-year cumulative incidence of brain metastases 33% with PCI versus 59% without PCI), the trial failed to show a survival benefit with the addition of PCI.

Thus, for patients with ES-SCLC who have good response to chemotherapy, both PCI and active surveillance with brain MRIs and early salvage are appropriate, and shared decision-making to evaluate risks and benefits of either approach is warranted. PCI reduces the incidence of brain metastases but at the cost of neurocognitive function and quality of life, and its survival benefit has been questioned in the context of improved brain surveillance imaging. A phase 3 trial (SWOG S1827, NCT04155034) is ongoing to evaluate MRI surveillance with and without PCI in both LS- and ES-SCLC.

For patients who do proceed with PCI, several strategies can be similarly employed as with WBRT to mitigate neurotoxicity. Extrapolating from the NRG-CC001 trial,^19^ memantine, and hippocampal-sparing radiation techniques can be considered. A Dutch randomized trial of hippocampal-sparing PCI (25 Gy in 10 fractions) for 168 patients with SCLC was recently published. In contrast with NRG-CC001, this trial did not demonstrate lower probability of cognitive decline with hippocampal-sparing technique as measured by the Hopkins Verbal learning test.^86^ Critiques of the trial have included its small sample size and limited power to detect small changes, and several other larger trials are still ongoing (NRG CC003, NCT02635009). Radiation dose and patient age should also be considered as factors impacting tolerability of PCI. The preferred dose of PCI is 25 Gy in 10 fractions, with higher doses associated with increased toxicity and mortality.^87^ Older patients are more vulnerable to PCI as 83% of patients older than 60 years developed chronic neurotoxicity at 1 year following PCI compared to 56% of patients younger than 60 on RTOG 0214.^88^

### Radiosurgery for SCLC

WBRT has long been considered standard therapy for SCLC brain metastases due to concern for rapid and diffuse intracranial progression. SCLC was excluded from many of the landmark trials showing superiority of SRS over WBRT in terms of cognitive function for patients with limited brain metastases.^14,15^ However, retrospective series have suggested that as with other histologies, SRS may be a reasonable alternative to WBRT for SCLC brain metastases with less adverse effect and no detriment to survival. One of the first studies looking at this retrospectively compared 34 patients with SCLC brain metastases to 211 patients with NSCLC brain metastases treated with radiosurgery.^89^ There was no difference between the two groups in terms of tumor control, neurological survival, or overall survival. Outcomes with first-line SRS for SCLC brain metastases were recently reported in a large international multicenter “First-line Radiosurgery for Small-Cell Lung Cancer Brain Metastases (FIRE-SCLC)” cohort study.^90^ This cohort included 710 patients treated with SRS at 28 centers in 6 countries between 1994 and 2018. Median overall survival and time to central nervous system progression were 8.5 and 8.1 months, respectively. One-year cumulative incidence of distant intracranial failure was 41.6%; of patients who received salvage treatment, 33.5% received salvage SRS and only 16.1% received salvage WBRT. Concerning outcomes that may be associated with omission of WBRT were uncommon, with these including leptomeningeal progression (10.8%) and neurological death (12.4%). Finally, the authors compared this cohort with individual patient data (219 patients) from a large published data set of first-line WBRT for SCLC using propensity-score matching. As expected, WBRT was associated with improved intracranial control, but without an improvement in overall survival.

With improved systemic therapy (eg, immunotherapy) and prognoses for patients with SCLC, the value of long-term neurocognitive preservation maybe even more important. Further, systemic therapy may help control microscopic intracranial disease that previously required WBRT. Based on the above accumulating data suggesting that SRS may be safe and efficacious for some patients with SCLC, there are several ongoing trials evaluating the role of SRS in SCLC. One of these includes a German randomized trial of WBRT versus SRS for 1–20 SCLC brain metastases (ENCEPHALON study, NCT03297788).

### CNS Activity of Systemic Therapy

Given the high risk of brain metastases in SCLC, systemic therapy plays a secondary role in radiation with respect to the management of intracranial disease. However, intracranial activity has been demonstrated in SCLC with regimens that include etoposide, irinotecan/topotecan, and temozolomide.^91–93^ In addition, the immune checkpoint inhibitors, atezolizumab, and durvalumab, are now approved for use in ES-SCLC in combination with cisplatin or carboplatin plus etoposide. However, trials leading to these approvals included only patients with asymptomatic and/or treated brain metastases, and determining whether the addition of immunotherapy changes the natural history of intracranial disease in SCLC requires further assessment.^94,95^

## Conclusion

The management of brain metastases in lung cancer requires a collaborative, multidisciplinary approach among radiation oncologists, medical oncologists, and neurosurgeons. Given the impact of brain metastases on both prognosis and quality of life, the effective coordination of local and systemic therapies remains a priority, especially as extracranial disease control in lung cancer improves. To fully optimize the tools currently available for treating intracranial metastases, further prospective studies are needed to evaluate the most effective combinations of local radiotherapy and novel systemic therapies. In the same way that systemic therapy for metastatic lung cancer has evolved from a one-size-fits-all approach to one that accounts for molecular and pathologic tumor features, the goal for intracranial management should be better understand how tumor mutations, immune markers, extent of intracranial disease burden, and other patient- and tumor-specific factors might inform clinical decision-making in the modern treatment era.
